# MSFANet: A Multi-Scale Feature Fusion Transformer with Hybrid Attention for Remote Sensing Image Super-Resolution

**DOI:** 10.3390/s25216729

**Published:** 2025-11-03

**Authors:** Jie Yu, Chengcheng Lin, Luyao Peng, Cheng Zhong, Hui Li

**Affiliations:** 1 Badong National Observation and Research Station of Geohazards, China University of Geosciences, Wuhan 430074, China; jieyu@cug.edu.cn (J.Y.); linchengcheng@cug.edu.cn (C.L.); luyaopeng@cug.edu.cn (L.P.); zhonglxm@cug.edu.cn (C.Z.); 2 The School of Earth Sciences, China University of Geosciences, Wuhan 430074, China

**Keywords:** remote sensing, super-resolution reconstruction, Swin Transformer, deep learning, attention mechanism

## Abstract

To address the issue of insufficient resolution in remote sensing images due to limitations in sensors and transmission, this paper proposes a multi-scale feature fusion model, MSFANet, based on the Swin Transformer architecture for remote sensing image super-resolution reconstruction. The model comprises three main modules: shallow feature extraction, deep feature extraction, and high-quality image reconstruction. The deep feature extraction module innovatively introduces three core components: Feature Refinement Augmentation (FRA), Local Structure Optimization (LSO), and Residual Fusion Network (RFN), which effectively extract and adaptively aggregate multi-scale information from local to global levels. Experiments conducted on three public remote sensing datasets (RSSCN7, AID, and WHU-RS19) demonstrate that MSFANet outperforms state-of-the-art models (including HSENet and TransENet) across five evaluation metrics in ×2, ×3, and ×4 super-resolution tasks. Furthermore, MSFANet achieves superior reconstruction quality with reduced computational overhead, striking an optimal balance between efficiency and performance. This positions MSFANet as an effective solution for remote sensing image super-resolution applications.

## 1. Introduction

Remote sensing images are widely used in fields such as urban planning, disaster assessment, and agriculture [1,2,3,4]. Spatial resolution—the smallest distinguishable ground feature—directly determines image detail. High-resolution imagery enables the identification of fine-grained details such as buildings and roads. In contrast, insufficient spatial resolution significantly compromises application accuracy [5]. For example, imagery with 10–30 m resolution cannot distinguish between residential and commercial zones [6] nor effectively monitor crop growth stages [7]. Similarly, features such as collapsed structures [8] and subtle geological formations like minor fault lines remain undetectable in low-resolution imagery [9]. Nevertheless, pursuing higher resolution faces two fundamental constraints: sensor hardware advancements demand expensive microfabrication processes [10], while increased data volumes strain transmission and storage infrastructures [10,11]. In this context, super-resolution technology emerges as a cost-effective alternative, enhancing image resolution algorithmically without hardware investment, mitigating data burdens, and ultimately improving data utility for fine-grained applications [9,10,11,12].

Initially, people primarily relied on methods based on interpolation and transform domains to enhance image resolution [13,14,15]. Common interpolation techniques include bilinear interpolation and bicubic interpolation. These methods offer the advantage of low computational complexity, enabling rapid image processing with strong real-time capabilities [16]. However, their ability to restore images is constrained by the quality of the original low-resolution image [17]. These methods often struggle to preserve the true structure and high-frequency details of an image, resulting in insufficient clarity and detail richness. Transform-domain methods, such as those based on wavelet domains and sparse representations, can enhance image details to some extent but also present limitations. These approaches may introduce blurring and excessive smoothing during image processing, posing significant challenges for tasks requiring fine details, such as object recognition [18,19]. While using multiple images can enhance overall resolution and capture more details, this method also introduces a series of challenges. For instance, issues like image alignment problems, increased computational complexity, data redundancy, and inconsistencies arising from temporal variations can all adversely affect the quality of image reconstruction [20,21,22,23].

Owing to its capacity to learn complex patterns, restore fine details, adapt to complex degradation processes, and enhance image quality, deep learning has been gaining increasing popularity in the field of image super-resolution reconstruction. Deep learning can effectively reduce common artifacts, blurring, or blocky effects that are often encountered in traditional methods. Since around 2014, convolutional neural networks (CNNs) have been widely applied in the field of image super-resolution. For instance, SRCNN [24], as an early representative work, achieved super-resolution reconstruction by learning the mapping relationship between low-resolution and high-resolution images. Subsequently, numerous improved CNN architectures have been proposed, such as VDSR [25], SRResNet [26], CFSRCNN [27], ESRGCNN [28], and LESRCNN [29], all of which have achieved significant performance improvements. CNNs are highly adaptable to various input data, including different types of remote sensing images, sensor conditions, and degradation processes. In addition, generative adversarial networks (GANs) have also achieved good results in super-resolution reconstruction. For example, SRGAN [30], by introducing adversarial training, is capable of generating more realistic high-resolution images, which not only shows improvement in objective metrics but also appears more natural in visual effects. Since then, improved methods such as ESRGAN [31], GAN-CIRCLE [32], SOUP-GAN [33], and CAL-GAN [34] have continued to emerge, further optimizing the texture details and visual quality of images and providing new ideas and methods for the development of the field of image super-resolution.

In recent years, the remarkable performance of large Transformer models in the field of natural language processing has spurred significant advancements in their application to computer vision. TransENet [35] leverages Transformer modules to capture long-range dependencies and effectively uncover correlations between high-dimensional and low-dimensional features. By integrating Transformer modules with convolutional layers, TransENet is capable of capturing fine-grained and comprehensive contextual information. Additionally, ESRT [36] and ESSAformer [37] have introduced Transformer architectures that have significantly enhanced the performance of single-image super-resolution, particularly excelling in handling complex textures and details. These contributions have provided innovative perspectives for research on Transformer-based image super-resolution.

In the realm of image super-resolution, non-Transformer architectures have also garnered significant achievements. HSENet [38], with its distinctive hierarchical structure, adeptly captures both global and local features across multiple scales. It excels not only in detail preservation but also ensures contextual consistency. OmniSR [39], through its innovative architecture and training strategy, achieves efficient image processing across diverse content types and degradation scenarios, thereby substantially enhancing the quality and detail of reconstructed images. HAUNet [40], via optimized network design and efficient feature extraction mechanisms, significantly boosts processing speed and resource efficiency in super-resolution while maintaining image quality. This renders it particularly suitable for real-time or resource-constrained applications. BSRAW [41] proposes a blind super-resolution method for RAW domain images. It designs realistic degradation pipelines specifically for training models to process raw sensor data. This approach effectively addresses common issues such as sensor noise, defocus, and exposure, thereby significantly enhancing the enlargement and quality of real-world RAW images. ASID [42], a lightweight super-resolution network, reduces computational overhead by refining information distillation and introducing cross-block attention sharing.

Recent research has shifted its focus from designing individual convolutional structures toward a synergistic integration of generative priors, efficient global dependency modeling, and realistic physical degradation simulation [43,44,45,46]. On one hand, diffusion-based generative methods have demonstrated significant advantages in reconstructing high-frequency textures and complex structural details through iterative denoising processes [47,48]. These approaches mitigate the over-smoothing issues commonly found in deterministic reconstruction models, creating new possibilities for generating optically realistic imagery with enhanced detail [49,50]. On the other hand, visual Transformer architectures—exemplified by Swin Transformer—have shown powerful capabilities in capturing long-range spatial dependencies and global contextual information inherent in remote sensing imagery by leveraging window-based self-attention mechanisms and hierarchical designs [51,52,53,54]. In parallel, advances in self-supervised and weakly supervised learning are further reducing the reliance on large-scale, accurately paired datasets [55,56].

In this study, we aim to further enhance the quality of reconstructed images while maintaining efficiency and reliability by developing a novel model that leverages the latest advancements in deep learning. Specifically, we propose a new model called MSFANet, which is based on the Swin Transformer architecture. This model integrates three key components: Feature Refinement Augmentation (FRA), Local Structure Optimization (LSO), and Residual Fusion Network (RFN). These components work together to effectively extract and adaptively aggregate multi-scale information ranging from local to global, thereby achieving superior reconstruction quality and efficiency. Our model has demonstrated remarkable performance in terms of reconstruction quality and efficiency across multiple benchmark datasets.

The remainder of this paper is organized as follows: Section 2 provides a detailed description of the proposed MSFANet framework. Section 3 presents the experimental datasets and results. Section 4 offers a comprehensive discussion and analysis of the findings. This paper concludes with Section 5, which summarizes the main conclusions and implications of this study.

## 2. Methods

**Figure 1:**
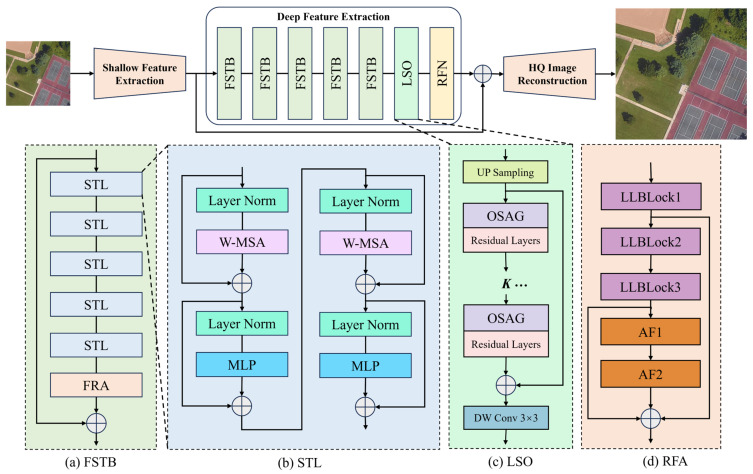
The network architecture of MSFANet. (**a**) FSTB: Feature Swin Transformer Block; (**b**) STL: Swin Transformer Layer; (**c**) LSO: Local Structure Optimization; (**d**) RFA: Feature Refinement Augmentation. In this and following figures, arrows indicate transmission medium and flow direction between components.

Inspired by SwinIR [55] and SwinFR [57], this paper proposes the MSFANet model based on the Swin Transformer architecture, whose structure is shown in Figure 1.

MSFANet comprises three key components: a shallow feature extraction module, a deep feature extraction module, and a high-quality image reconstruction module. The shallow feature extraction module and HQ image reconstruction module adopt the proven configuration from SwinFR to ensure model stability and effectiveness in both foundational feature extraction and final image reconstruction stages. Within the deep feature extraction module, MSFANet introduces a Feature Swin Transformer block (FSTB). Building upon the Swin Transformer layers rom SwinIR, this module incorporates our independently designed FRA (Figure 1a,d). This design significantly enhances the model’s hierarchical feature extraction and fusion capabilities. By emphasizing key feature weights, it enables the model to accurately capture both local details and global contextual information in remote sensing images, providing robust feature representation for high-quality super-resolution reconstruction. We employed a 5-layer FSTB architecture (5 STLs per layer) based on experimental findings. Additionally, MSFANet incorporates an LSO (Figure 1c) module. This module simulates a global self-attention mechanism through local convolutional operations, enhancing feature representation capabilities while effectively reducing computational complexity. Concurrently, MSFANet employs an RFN module that integrates dense residual learning with channel attention mechanisms. This module efficiently extracts, refines, and aggregates multi-level features from remote sensing images, providing rich and high-quality feature support for super-resolution reconstruction.

Remote sensing imagery exhibits multi-scale heterogeneity, where macroscopic scene structures (such as urban blocks, watershed basins) coexist with microscopic sparse details (such as individual buildings, narrow roads). Furthermore, the importance of different features (e.g., multispectral bands or deep semantic channels) varies significantly across channel dimension. Pure spatial attention mechanisms focus solely on pixel-level correlations and fail to capture the value differences among spectral channels—for instance, the unique value of the near-infrared band for vegetation analysis versus the role of visible light bands in structural identification. While pure self-attention mechanisms can capture global dependencies, their computational complexity increases quadratically with image size, making them impractical for processing large-scale remote sensing data. To overcome these limitations, MSFANet introduces a tailored hybrid attention mechanism, with each component strategically designed to address specific bottlenecks in remote sensing image super-resolution.

### 2.1. Feature Refinement Augmentation

**Figure 2:**
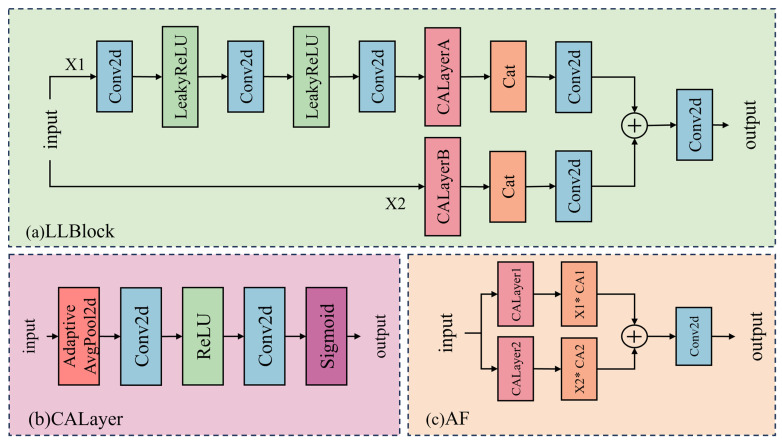
Detailed structure of the FRA block. (**a**) LLBLock: Lightweight Lattice Block; (**b**) CALayer: Channel Attention Layer; (**c**) AF: Attention Fusion module. In subfigure (**c**), the asterisk (*) symbolizes the element-wise multiplication operation performed between the feature tensor and its respective channel attention output.

As depicted in Figure 2, the FRA is one of the core components of MSFANet. It is designed to enhance hierarchical feature extraction and fusion, thereby enabling the model to effectively capture both local details and global context in remote sensing images. The FRA module comprises three key structures: the Lightweight Lattice Block (LLBlock), the Channel Attention Layer (CALayer), and the Attention Fusion (AF) module.

These components work in concert to optimize feature representation. The LLBlock is inspired by lattice filter banks. Upon receiving an input tensor, it first decomposes it into two sub-tensors $x_{1}\in R^{C_{1}\times H\times W}$ and $x_{2}\in R^{C_{2}\times H\times W}$. Subsequently, each sub-tensor is processed through three layers of 3 × 3 convolutional (Conv2d) operations, followed by LeakyReLU activation functions. This sequence of operations refines the deep features of each sub-tensor. The LLBlock employs a divide-and-conquer strategy to perform targeted feature extraction and enhancement on distinct sub-tensors, thereby laying the groundwork for subsequent feature fusion. The CALayer (as shown in Figure 2b) is designed to enhance key features. For the outputs $x_{1}^{\prime }$ and $x_{2}^{\prime }$ from the LLBlock, the CALayer calculates their respective channel attention weights. Specifically, it first applies global average pooling to compress the spatial dimensions, reducing the width and height dimensions of the feature map to 1 while preserving the global information in the channel dimension. Subsequently, two layers of 1 × 1 convolutions perform channel-wise feature extraction and transformation on the compressed features. Finally, a Sigmoid activation function generates a weight map. This weight map is then multiplied element-wise with the original feature map, thereby amplifying features in important channels while suppressing redundant information. This process achieves precise enhancement of key features. The AF (Figure 2c) module is responsible for multi-scale feature integration. For the initially split sub-tensors $x_{1}$ and $x_{2}$, the AF module applies the CALayer for weighted processing. This ensures efficient fusion of features across different levels. By doing so, the AF module not only preserves the richness of multi-scale features but also further optimizes feature quality through the channel attention mechanism. This high-quality feature representation provides robust support for super-resolution reconstruction tasks, thereby significantly enhancing the model’s performance in remote sensing image super-resolution reconstruction.

### 2.2. Local Structure Optimization

**Figure 3:**
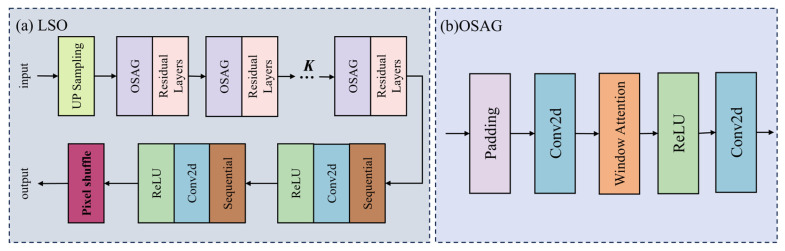
Detailed structure of the LSO block. (**a**) LSO: Local Structure Optimization; (**b**) OSAG: Overlapping Self-Attention Group.

As depicted in Figure 1c and Figure 3a, the LSO module is a pivotal component within the MSFANet architecture, tasked with enhancing the representation of local features in remote sensing images while balancing the precision of feature learning with computational efficiency. Functionally, the LSO module initiates the process by employing specialized convolutional layers to adjust the channel dimension of the input feature maps. This adjustment aligns the feature channels with the preset network processing dimensions, establishing a standardized feature input foundation for subsequent local structure optimization. By doing so, it prevents feature information loss or redundant computation due to channel dimension mismatch, ensuring that subsequent operations can focus efficiently on feature content optimization rather than dimension adaptation.

At the core of the LSO module lies the Overlapping Self-Attention Group (OSAG) module (Figure 3b), which simulates the local feature correlation capture capability of global self-attention mechanisms. The OSAG module employs dynamic window partitioning and padding strategies, adaptively calculating the padding amount based on the spatial dimensions of the input feature maps. This approach ensures that edge regions of the feature maps are processed with the same window scale as the central regions, effectively avoiding the loss of edge details that can occur with traditional fixed-window processing. By stacking convolutions with non-linear activation operations, the OSAG module accurately models the dependencies between pixels within local windows, enhancing the learning of contour and detail features of fine targets in remote sensing images, such as small buildings, road edges, and vegetation textures. This capability compensates for the limitations of traditional convolutional networks in local fine feature extraction, providing richer local structural information support for super-resolution reconstruction. Moreover, the LSO module incorporates a residual connection mechanism that fuses the initially adjusted feature maps with those optimized by the OSAG module. This fusion retains the global structural information of the original features while integrating the refined local features, preventing feature degradation that can occur during deep network training. It enhances the stability of model training and ensures that the global scene structure is not compromised by local optimizations, achieving a super-resolution effect where “global structure is coherent, and local details are clear”.

Additionally, the LSO module utilizes a PixelShuffle layer to perform upsampling of the feature maps, mapping the optimized local features from the low-resolution space to the target high-resolution space. Precise dimension cropping ensures that the output feature maps match the spatial dimensions of the target resolution for the super-resolution task. This provides directly usable high-resolution local features for subsequent image reconstruction stages.

### 2.3. Residual Feature Network

**Figure 4:**
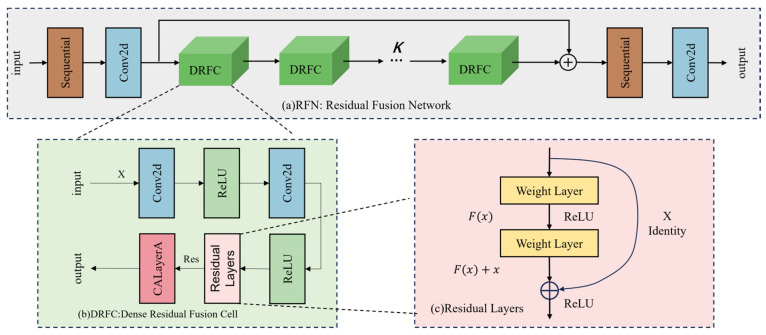
Detailed structure of the RFN block. (**a**) RFN: Residual Fusion Network; (**b**) DRFC: Dense Residual Fusion Cell; (**c**) Residual Layers.

Figure 4 illustrates the detailed structure of the Residual Fusion Network (RFN) block in MSFANet.

As illustrated in Figure 4a, the RFN module is a central component within the MSFANet architecture, tasked with the efficient extraction, refinement, and aggregation of multi-level features from remote sensing images. Functionally, the RFN module begins by employing specialized convolutional layers to perform initial encoding of the input features. It transforms the diverse features originating from the FSTB and the LSO module into a unified dimensional initial feature representation. This standardized input foundation is crucial for subsequent deep feature learning, as it prevents information discontinuities or redundancies that might arise from feature source disparities, ensuring that multi-level features can be effectively integrated within the same processing framework.

The RFN module’s innovative strength lies in the introduction of the Dense Residual Fusion Cell (DRFC), as shown in Figure 4b, and the dynamic configuration of its quantity. Each DRFC integrates convolutional operations, non-linear activation, and channel attention mechanisms. The convolutional layers are designed to capture feature details at various scales, such as architectural textures, vegetation distribution, and water body boundaries in remote sensing images. Non-linear activation functions enhance the model’s ability to fit complex feature patterns, while the channel attention mechanism quantifies the importance of each feature channel, adaptively reinforcing channels that contain high-frequency details and structural information, and suppressing interference from noise and background channels. Furthermore, the DRFC employs a dense connection design, allowing each cell to directly receive the output features of all preceding cells. This not only maximizes the utilization of complementary information across different feature levels but also prevents the common issue of feature attenuation in deep networks. The residual propagation within the DRFC helps mitigate the vanishing gradient problem, enhancing the stability and convergence efficiency of model training, thus providing ample deep feature support for super-resolution tasks. In the feature output phase, the RFN module uses a final convolutional layer to map the deep features processed by the DRFC to the target super-resolution output space, facilitating the transformation from abstract features to high-resolution image features. During this process, the aggregation effect of multi-level features is fully leveraged—shallow features provide basic texture and contour information, while deep features supplement fine structures and high-frequency details. The synergistic action of these features ensures that the super-resolution results maintain the structural coherence of the global scene while accurately restoring the details of local fine targets, such as small infrastructure and sparse vegetation in remote sensing images.

## 3. Experiment and Results

### 3.1. Dataset

In this study, the performance of the proposed method was evaluated using three publicly available remote sensing datasets: RSSCN7 [58], AID [59], and WHU-RS19 [60]. These datasets serve as critical benchmarks for training and evaluating deep learning models in various remote sensing applications, including classification, segmentation, scene recognition, object detection, and super-resolution tasks.

The RSSCN7 dataset, developed by the Institute of Remote Sensing and Digital Earth, Chinese Academy of Sciences, contains 2800 high-resolution images across seven classes: agriculture, commercial, forest, industrial, residential, river, and wasteland. Each class comprises 400 images (400 × 400 pixels), captured under varying seasonal, lighting, and platform conditions, ensuring high diversity and complexity. The AID dataset, created by Wuhan University, includes ~3000 high-resolution images spanning 30 land-use categories, such as airports, bridges, commercial areas, forests, and farmland. Each category contains 220–420 images (600 × 600 pixels), providing broad coverage of urban and natural scenes. The WHU-RS19 dataset, also from Wuhan University, consists of 950 high-resolution images divided into 19 categories, including airports, ports, bridges, industrial zones, forests, and sports fields. Each category has 50 images (600 × 600 pixels), sourced primarily from Google Earth, with diverse scenes and complex backgrounds.

All three datasets were partitioned into training, validation, and test sets using a consistent 70%/15%/15% ratio. Throughout this process, we strictly adhered to a category-balanced stratified random sampling strategy: samples were first grouped by category, followed by proportional random sampling within each group. This approach ensures that the category distributions across training, validation, and test sets remain fully consistent with the complete dataset, effectively mitigating experimental bias caused by data skew. Low-resolution (LR) images were synthesized from the original high-resolution (HR) images through bicubic interpolation downsampling. The downsampling factors strictly followed the specifications of the three super-resolution reconstruction tasks (×2, ×3, and ×4). This synthesis strategy effectively mitigates confounding factors inherent in real remote sensing scenarios—such as sensor noise, atmospheric scattering, and spatio-temporal misalignment—enables strict control over experimental variables and helps validate the core innovations [38,39,40,41,42,43,44,45,46,47,48,49,50,51,52,53,54,55].

### 3.2. Performance Assessment

We compared our proposed method with nine SOTA models, including VDSR, SRDD [61], and OmniSR for natural images, as well as HSENet, TransENet, FENet [62], LGCNet [63], BSRAW and ASID which were specifically designed for RSIS tasks. All comparison methods were retrained using open-source code and tested under the same conditions. We generated low-resolution images from the high-resolution images in the datasets using bicubic interpolation to train the models, while the original high-resolution images were used for model validation. We employed five metrics to comprehensively evaluate the quality of the reconstructed images, including the Peak Signal-to-Noise Ratio (PSNR), Structural Similarity Index (SSIM), Spatial Correlation Coefficient (SCC), Spectral Angle Mapper (SAM), and Universal Quality Index (UQI).

PSNR is a widely used objective metric to evaluate the quality of an image after it has undergone some form of transformation, such as compression, noise reduction, or fusion. It quantifies the level of distortion by comparing the original (reference) image to the reconstructed image. A higher PSNR value indicates better quality, as it signifies a closer match between the reconstructed image and the original.

(1) $$PSNR=10\cdot \log_{10}\left(\frac{MAX_{i}^{2}}{MSE}\right)$$

(2) $$MSE=\frac{1}{N_{W}N_{H}}\sum_{i=0}^{N_{W}-1}\;\sum_{j=0}^{N_{H}-1}\;{\left[Y\left(i,j\right)-X\left(i,j\right)\right]}^{2}$$

SSIM is a measure of the similarity of the two images, to evaluate image fidelity after transformations. Unlike PSNR, which focuses solely on pixel-level error, SSIM considers perceptual factors, making it more aligned with human visual perception.

(3) $$SSIM\left(X,Y\right)=\frac{\left(2\mu_{X}\mu_{Y}+C_{1}\right)\left(\sigma_{XY}+C_{2}\right)}{\left(\mu_{X}^{2}+\mu_{Y}^{2}+C_{1}\right)\left(\sigma_{X}^{2}+\sigma_{Y}^{2}+C_{2}\right)}$$

SCC focuses on the spatial correlation between images, making it sensitive to spatial relationships rather than just pixel intensity differences. It is particularly useful for assessing the alignment or similarity of spatial structures, which is important in image processing applications such as image fusion, registration, and super-resolution.

(4) $$SCC=\frac{\sum_{i,j}\;\left(I_{ij}-\bar{I}\right)\left(R_{ij}-\bar{R}\right)}{\sqrt{\sum_{i,j}\;(I_{ij}-\bar{I}{)}^{2}\sum_{i,j}\;(R_{ij}-\bar{R}{)}^{2}}}$$

SAM is frequently used in multispectral and hyperspectral image analysis to measure the spectral similarity between each pixel in a reference image and the corresponding pixel in a reconstructed image. SAM is effective for assessing how well spectral features are preserved during processes such as image fusion, compression, and enhancement.

(5) $$SAM\left(X,Y\right)={cos}^{-1}\left(\frac{X\cdot Y}{\left\|X\right\|\left\|Y\right\|}\right)$$

UQI is an image quality assessment metric that measures the similarity between two images by considering structural, luminance, and contrast aspects. UQI focuses on structural similarity rather than just pixel-level differences.

(6) $$UQI\left(X,Y\right)=\frac{{4\cdot \mu }_{X}\mu_{Y}\cdot \sigma_{XY}}{\left(\mu_{X}^{2}+\mu_{Y}^{2}\right)\left(\sigma_{X}^{2}+\sigma_{Y}^{2}\right)}$$

where Y denotes the reconstructed image, X denotes the reference image, $\sigma_{X}$ and $\sigma_{Y}$ represent the standard deviation of X and Y, $\sigma_{XY}$ represents the covariance in X and Y, while $\left\|X\right\|$ and $\left\|Y\right\|$ denote the magnitudes (or norms) of the spectral vectors X and $Y{,\;\sigma }_{XY}$ denotes the covariance between X and Y.

### 3.3. Experimental Setting

In this research, we conduct a thorough assessment of the proposed model’s performance across ×2, ×3, and ×4 super-resolution tasks, benchmarking it against a selection of cutting-edge (SOTA) efficient models. All comparison methods were retrained using open-source code and tested under the same conditions. The training batch size for all tasks is uniformly set to 32. The model was optimized using the Adam optimizer with parameters β_1_ = 0.9, β_2_ = 0.99, and ε = 1 × 10^8^. The initial learning rate was set to 1 × 10^4^ and gradually reduced to 1 × 10^6^ over 300 training epochs using a cosine annealing scheme (Cosine Annealing LR). The model was implemented in PyTorch 2.1.0 and all experiments were conducted on a workstation equipped with two NVIDIA GeForce GTX 3090 GPUs (NVIDIA Corporation, Santa Clara, CA, USA).

### 3.4. Comparison with Other Models

We evaluated the performance of our proposed model across ×2, ×3, and ×4 super-resolution tasks, comparing it with VDSR [25], SRDD [61], HSENet [38], TransENet [35], FENet [62], LGCNet [63], OmniSR [39], BSRAW [41], and ASID [42] on the RSSCN7 [58], AID [59], and WHU-RS19 [60] datasets. The results of these comparisons are detailed in Table 1, Table 2 and Table 3 and Tables S1–S3.

The MSFANet model outperforms all SOTA models on the RSSCN7 dataset (Table 1). Specifically, under ×2 magnification processing, MSFANet achieves the highest PSNR (28.29 dB), SSIM (0.7683), and UQI (0.9921). In experiments on the RSSCN7 dataset, our proposed MSFANet demonstrated outstanding performance at ×2, ×3, and ×4 magnification levels. At ×2 magnification, MSFANet achieved a PSNR value of 28.29 dB, surpassing ASID (28.23 dB) and BSRAW (28.21 dB) by 0.21% and 0.28%, respectively. Simultaneously, its SSIM value reached 0.7683, surpassing ASID (0.21%) and BSRAW (0.33%). At ×3 magnification, MSFANet’s PSNR was 26.52 dB, outperforming ASID (26.38 dB) and BSRAW (26.31 dB). Its SSIM value of 0.6714 was 0.68% and 1.49% higher than ASID and BSRAW, respectively. At ×4 magnification, MSFANet achieved a PSNR of 25.48 dB, surpassing ASID (25.35 dB) and BSRAW (25.30 dB). Its SSIM value reached 0.6093, representing improvements of 0.88% and 1.33% over ASID and BSRAW, respectively. Furthermore, MSFANet consistently outperformed ASID and BSRAW on the SCC metric across all magnification levels. Specifically, at ×2, ×3, and ×4 magnification, MSFANet’s SAM values improved by 1.09%, 2.84%, and 1.54% over ASID, and by 1.38%, 3.76%, and 2.10% over BSRAW. Compared to traditional algorithms like VDSR and SRDD, MSFANet demonstrates more significant improvements. At ×2 magnification, MSFANet achieves PSNR values 0.86% and 1.09% higher than VDSR and SRDD, respectively.

As illustrated in Table S1, the MSFANet model demonstrates exceptional performance across diverse categories within the RSSCN7 dataset for the ×4 upscaling task, consistently achieving the highest PSNR and SSIM values in the majority of these categories. Specifically, MSFANet attains a PSNR of 28.34 dB in the “Grasslands” category, 30.63 dB in the “Forests” category, and 26.94 dB in the “Parking Lots” category. Additionally, it secures the top SSIM values with 0.6756 in “Grasslands,” 0.6337 in “Forests,” and 0.7362 in “Parking Lots”. In the “Village” and “Factory” categories, MSFANet also shows strong competitive performance, recording PSNR values of 24.77 dB and 21.88 dB, and SSIM values of 0.4736 and 0.5787, respectively. On average, MSFANet achieves an impressive PSNR of 25.48 dB and an SSIM of 0.61, underscoring its superior capabilities in image super-resolution tasks when compared to other models.

Table 2 and Table 3 showcase the superior performance of the MSFANet model across various metrics and scales on both the AID and WHU-RS19 datasets. The model consistently achieves the highest scores in PSNR, SSIM, SCC, SAM, and UQI, highlighting its exceptional capabilities in terms of image quality, structural coherence, and detail preservation. Tables S2 and S3 provide a detailed analysis of various scenes within the AID and WHU-RS19 datasets, further confirming that the MSFANet model delivers the most balanced performance. This well-rounded efficacy lays a solid foundation for remote sensing image super-resolution reconstruction.

**Figure 5:**
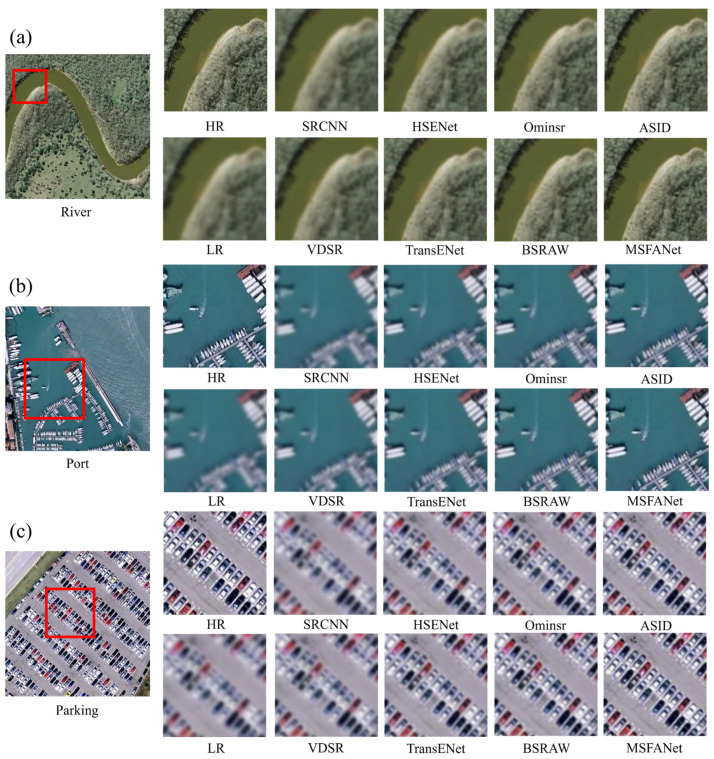
Comparative visualization on the RSSCN7 dataset. (**a**) River, (**b**) Port, (**c**) Parking lot. The red boxes highlight the focus regions for illustration.

Figure 5 demonstrates that the MSFANet algorithm shows outstanding performance in super-resolution reconstruction in various scenarios and surpasses other algorithms, especially in accurately capturing and restoring complex image details. In the river image (Figure 5a), MSFANet creates a clearer and sharper portrayal of the riverbank textures and contours, leading to a higher-resolution output. For the port scene (Figure 5b), the algorithm effectively distinguishes the port structure and its boundary with the surrounding water, presenting more intricate details. In the parking lot image (Figure 5c), MSFANet achieves more precise reconstruction of the vehicle shapes, parking space markings, and surrounding roads. Figures S1 and S2 also illustrate the excellent super-resolution effects of MSFANet.

### 3.5. Ablation Study

This ablation study systematically assesses the influence of various parameter configurations on model efficacy, with a focus on Memory, PSNR, and SSIM as principal metrics of evaluation. The analysis was executed across three distinct remote sensing datasets: RSSCN7, AID, and WHU-RS19, encompassing a total of eight experimental setups denoted as L0 through L7.

Table 4 summarizes the performance comparison of various model configurations on image processing tasks in terms of parameter count, memory consumption, and PSNR/SSIM metrics. The baseline model (L0) features a low parameter count (1.37 M) and memory footprint (5.26 MB), but delivers relatively poor PSNR and SSIM results across all three datasets. For instance, on the RSSCN7 dataset, it achieves a PSNR of 24.84 and an SSIM of 0.5738. As additional modules are incorporated, both performance and resource requirements evolve. The FRA (L1) and LSO (L2) modules introduce only marginal increases in parameters and memory, while providing limited performance gains. In contrast, the RFN module (L3) substantially increases the parameter count (8.93 M) and memory consumption (12.10 MB), accompanied by noticeable performance improvements. Combined configurations—FRA+LSO (L4), FRA+RFN (L5), and LSO+RFN (L6)—achieve varying trade-offs between performance and resource cost. The full model, MSFANet (L7), which integrates all three modules, attains the highest parameter count (11.98 M) and memory usage (16.3 MB), but also yields the best PSNR and SSIM results across all datasets: on RSSCN7, PSNR = 25.48, SSIM = 0.6093; on AID, PSNR = 29.1, SSIM = 0.7848; and on WHU-RS19, PSNR = 26.75, SSIM = 0.6873. Specifically, compared to the baseline, MSFANet achieves approximate improvements of 2.64 in PSNR and 0.0355 in SSIM on RSSCN7; 1.46 in PSNR and 0.054 in SSIM on AID; and 0.8 in PSNR and 0.0379 in SSIM on WHU-RS19. These results demonstrate that MSFANet significantly enhances reconstruction quality through the integration of multiple modules, despite the increased computational cost. The fusion architecture effectively captures fine image details and structural information, enabling high-quality outputs and underscoring its strong suitability for complex image processing tasks.

In this study, MSFANet is developed based on the Swin Transformer architecture. To determine the optimal window size and attention head count for super-resolution tasks in remote sensing imagery, we systematically evaluated the impact of varying window sizes (2 × 2, 4 × 4, 8 × 8, and 16 × 16) and attention head counts (2 and 4) on model performance. MSFANet served as the baseline model, and experiments were conducted across two benchmark remote sensing datasets: AID and WHU-RS19. Multiple comparative experiments were performed, with PSNR and SSIM as the primary evaluation metrics (Table 5). The results demonstrate that both window size and attention head count significantly influence model performance, with the optimal configuration achieved at a window size of 8 × 8 and 4 attention heads. Additionally, the analysis revealed that increasing the number of attention heads generally improves PSNR and SSIM values but incurs higher memory consumption. Based on these findings, all subsequent experiments in this study adopted a window size of 8×8 and 4 attention heads for MSFANet to balance performance and computational efficiency.

### 3.6. Hardware Resource Consumption Analysis

**Figure 6:**
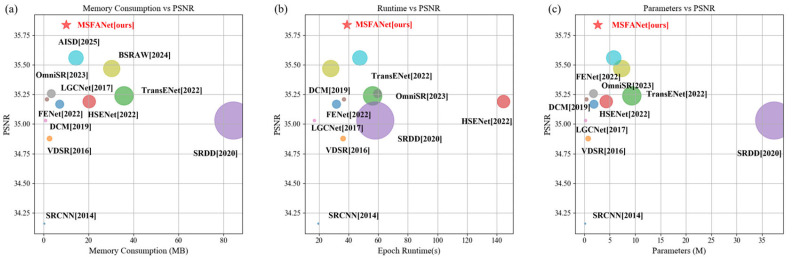
Computational resource analysis of models on AID dataset at 2× upscaling. (**a**) Memory consumption versus PSNR, (**b**) runtime versus PSNR, and (**c**) parameter count versus PSNR. Here, colorful circles denote the SOTA models, while the red star represents the proposed MSFANet model. Larger markers indicating higher consumption and smaller ones reflecting greater efficiency. Conventional CNN architectures (SRCNN, VDSR) are rendered in light blue, while Transformer-based approaches (TransENet, ASID) appear in medium blue.

In addition to evaluating reconstruction quality, we conducted comparisons of hardware resource consumption across models (Figure 6). Compared to SOTA models, MSFANet achieves superior performance with lower memory usage (Figure 6a). Specifically, it reduces memory consumption by 56.6% versus ASID and 29.75% versus BSRAW, while simultaneously improving PSNR by 0.25 dB over BSRAW and 0.18 dB over ASID. When compared to lightweight models (e.g., SRCNN, VDSR, DCM), MSFANet maintains a slight memory overhead but delivers significant PSNR improvements (>0.75 dB). As evidenced by Figure 6b,c, MSFANet also demonstrates advantages in both parameter efficiency and computational speed, requiring fewer computational resources while achieving better super-resolution outcomes. These results collectively validate the model’s exceptional efficiency-performance balance.

Unlike ASID’s “Attention Distillation,” which solely optimizes computational efficiency, MSFANet forms a closed-loop process of “selection-optimization-fusion” through FRA-enhanced multi-scale feature selection, LSO-reduced local computational costs, and RFN-mitigated feature decay. This achieves dual improvements in accuracy and efficiency. MSFANet achieves dual improvements in accuracy and efficiency by forming a closed-loop process of ‘selection-optimization-fusion’. This is accomplished through FRA to enhance multi-scale feature selection, LSO to reduce local computational costs, and RFN to mitigate feature decay.

## 4. Discussion

### 4.1. Advances in Other Remote Sensing Applications

While the current study focuses on remote sensing super-resolution improvement, recent advances in other domains are also driving the field toward greater intelligence and precision, thereby facilitating the in-depth exploration and application of geospatial knowledge.

In the domain of remote sensing image classification, cross-modal cross-attention Transformers, built upon the Transformer architecture, have significantly enhanced the performance of land cover classification and target recognition tasks by effectively integrating multimodal remote sensing data. Representative approaches such as the MFT [64] model propose an mCrossPA mechanism, which treats image patches from different modalities as queries and keys/values for cross-attention computation, enabling efficient multimodal fusion. The MCAITN [65] model introduces a multi-feature cross-attention induction mechanism, combining it with a Transformer backbone to fuse multi-scale features of hyperspectral images, demonstrating excellent performance in tasks like vegetation type identification and soil classification. Furthermore, models like CCFormer [66] and CM-Net [67] have validated the superiority of this mechanism on benchmark datasets such as Houston and Trento.

In the field of anomaly detection, memory-augmented autoencoders with adaptive reconstruction enhance the reconstruction of normal samples while suppressing the reconstruction of anomalous regions under unsupervised conditions [68]. This makes them suitable for tasks such as land cover anomaly identification and disaster monitoring in hyperspectral remote sensing images. The MAAE [69] model proposes superpixel-guided adaptive weight computation, sample affiliation mining, and entropy-based sparse addressing mechanisms, significantly improving anomaly detection capabilities in complex backgrounds. Meanwhile, the MAENet [70] model enhances the generalization and robustness of the model in noisy data, making it applicable to scenarios such as mineral exploration and disaster assessment.

For change detection tasks, dual-domain attention models effectively capture fine-grained change information in bi-temporal remote sensing images, such as building expansion and land cover type changes, by integrating spatial and frequency domain features. Representative methods include D^2^Former [71], which employs a hybrid CNN-Transformer architecture with a U-Net-style encoder-decoder to extract multi-scale features; DDCDNet [72], which uses a weight-shared Swin Transformer encoder for semantic feature extraction; and models like FTransDF-Net [73] and DML-UNet [74], which further demonstrate diverse implementations and application potential of this mechanism in change detection.

### 4.2. Potential Applications and Challenges

MSFANet’s capability to reconstruct high-quality, high-resolution images from low-resolution inputs through algorithmic processing endows it with broad and profound application potential across numerous fields. It represents not merely a simple enhancement in image quality, but a key to unlocking more detailed and accurate geospatial information, thereby driving paradigm shifts in scientific research, commercial applications, and public services.

High-resolution foundational geospatial products form the cornerstone of national economic and social development. MSFANet can effectively compensate for the resolution deficiencies in historical archives or partially updated contemporary data. For large-scale mapping projects, acquiring sub-meter imagery across entire regions is both costly and time-consuming. By enhancing widely available medium-resolution imagery (such as Landsat and Sentinel series), MSFANet can generate base maps with enriched details, accelerating the production and updating of global-scale 1:5000 or larger-scale maps—particularly beneficial for remote areas lacking current surveying and mapping data.

In Earth observation applications, details often directly determine decision-making accuracy [1,2,5,6]. MSFANet enables the identification of small agricultural plots, irrigation channels, and even early signs of crop stress (such as initial pest and disease patches) that are indistinguishable in medium-resolution imagery. This supports more scientific decisions in variable-rate fertilization and precision irrigation, simultaneously increasing yields while reducing agrochemical use, thereby promoting sustainable agricultural development. The technology also aids in precise tree species distribution mapping, monitoring small-scale illegal deforestation, and assessing forest health conditions (such as canopy loss). In biodiversity conservation, it can even assist in identifying habitat ranges and behavioral patterns of large wildlife. Furthermore, it enables more accurate delineation of water body boundaries, monitoring subtle water volume changes in reservoirs and lakes, and identifying detailed distribution of algal blooms caused by eutrophication, providing early warnings for water resource management and pollution control [8,9,10,11,12].

During disasters and crises, high-resolution imagery translates into invaluable decision-making time and more effective response measures [1,2,3,4,5,6]. Following major natural disasters like floods, earthquakes, or wildfires, weather conditions or smoke often compromise imaging quality, resulting in suboptimal resolution. MSFANet can rapidly reconstruct clearer visualizations, helping rescue forces accurately locate damaged buildings, road disruptions, and temporary settlements of affected populations, significantly enhancing rescue efficiency. In national and public security domains, the super-resolution technology can enhance low-quality imagery from surveillance areas, assisting in identifying key targets, vehicle models, or vessel characteristics. In urban management, it also helps identify governance “blind spots” such as illegal constructions and unauthorized waste disposal sites.

In scientific research, by enhancing historical remote sensing data (such as Landsat imagery from decades ago), MSFANet enables the establishment of longer-term, more detailed records of glacier retreat, coastline changes, and polar ice melt, providing more accurate validation data for climate change models. It allows researchers to analyze urban morphology at finer scales—examining relationships between building density and energy consumption, correlations between neighborhood layouts and heat island effects, and impacts of green space distribution on resident health—thereby advancing theories in urban planning.

Although the technical aspects of the model have been thoroughly covered, MSFANet faces several challenges in practical deployment. The first challenge is computational resource constraints, particularly for deployment on edge devices. Although MSFANet reduces computational complexity through its hybrid attention mechanism, further optimization is still required when processing extremely large remote sensing images. Potential solutions include developing adaptive computation pathways that dynamically allocate computational resources based on image content complexity, or designing hierarchical super-resolution strategies that apply different reconstruction precision to regions of varying importance. The second challenge involves domain adaptation issues. In real-world application scenarios, distribution discrepancies between training and testing data can significantly impact model performance. This necessitates strong cross-domain generalization capabilities. Future work could explore strategies based on meta-learning or domain adaptation to enable MSFANet to quickly adapt to new remote sensing environments and data distributions. The third challenge lies in the super resolution model with other remote sensing processing tasks. Remote sensing analysis typically involves multi-task collaborative workflows, making the seamless integration of MSFANet into complete “super-resolution—classification—detection—change analysis” pipelines a critical issue to address. We plan to develop jointly optimized frameworks that enable end-to-end training of super-resolution with other tasks, thereby maximizing overall performance.

### 4.3. Future Works

Efficiency and lightweight design are pivotal for deploying models in practical scenarios, particularly on edge devices. Neural Architecture Search (NAS) can be employed to automatically discover optimal network architectures under specific computational constraints (e.g., latency and memory usage), replacing manual design to achieve an optimal balance between performance and efficiency. The implementation of “early-exit” mechanisms or conditional computation pathways enables the model to dynamically allocate computational resources based on the complexity of image regions (e.g., employing simplified processing for flat areas and more intensive computation for texture-rich regions). Furthermore, model pruning, quantization, and knowledge distillation methods tailored for super-resolution tasks should be explored. For instance, channel-level pruning strategies can eliminate redundant feature channels, while knowledge distillation allows a compact student model to approximate the performance of a larger teacher network.

Moving beyond conventional metrics such as PSNR and SSIM, future efforts should prioritize achieving visually realistic and physically plausible results. The integration of diffusion models can help capture the distribution of high-frequency details, generating richer and more authentic textures while mitigating issues like GAN mode collapse and training instability. This approach effectively alleviates the over-smoothing artifacts commonly associated with deterministic models. In the context of remote sensing imagery, it is crucial to preserve the spectral characteristics of ground objects during reconstruction. Future models should incorporate measures such as the Spectral Angle Mapper (SAM) as part of the loss function to ensure that reconstructed outputs are suitable for subsequent quantitative remote sensing analysis, rather than merely enhancing visual appearance. Additionally, leveraging large-scale pre-trained semantic segmentation networks (e.g., CLIP) to construct perceptual losses can ensure semantic consistency in reconstructed images and prevent structurally implausible object representations.

Real-world image degradation is far more complex than simple bicubic downsampling. Future research should shift its focus from non-blind super-resolution to blind super-resolution, developing models capable of handling unknown, complex, and potentially spatially varying degradation kernels, as well as real-world scenarios involving mixed noise, blur, and compression artifacts. This necessitates the use of generative adversarial networks or physical models to simulate more realistic degradation processes for training. Reformulating the super-resolution problem as a maximum a posteriori (MAP) estimation task and incorporating plug-and-play frameworks that combine deep learning-based denoising priors with iterative optimization algorithms can further enhance model robustness and adaptability.

## 5. Conclusions

This study proposes MSFANet, a multi-scale feature fusion Transformer with hybrid attention, designed to address the insufficient spatial resolution of remote sensing images caused by sensor limitations, transmission constraints, and external interference. Extensive experiments on three public remote sensing datasets (RSSCN7, AID, and WHU-RS19) for ×2, ×3, and ×4 super-resolution tasks demonstrate the superiority of MSFANet. It outperforms eight state-of-the-art models, including SRCNN, VDSR, ASID, and BSRAW, across five evaluation metrics. For example, on the RSSCN7 dataset at ×2 scaling, MSFANet achieves a PSNR of 28.29 dB and an SSIM of 0.7683, surpassing ASID by 0.21% in both metrics. Ablation studies confirm that FRA, LSO, and RFN work synergistically to enhance performance, with the full MSFANet (L7) configuration yielding the best results. In terms of hardware efficiency, MSFANet reduces memory consumption by 56.6% compared to ASID and by 29.75% compared to BSRAW while maintaining higher reconstruction quality. Overall, MSFANet effectively balances efficiency and performance, offering a reliable solution for remote sensing image super-resolution and establishing a foundation for its application in domains such as urban planning and disaster assessment.

## Figures and Tables

**Table 1:** The performance of the proposed model on the RSSCN7 dataset. The best results are highlighted in bold. The units for PSNR and SAM are dB and rad, respectively.

Scale	Metric	VDSR	SRDD	HSENet	TransENet	FENet	LGCNet	OmniSR	BSRAW	ASID	MSFANet
**×2**	**P** **S** **NR**	28.05	27.98	28.22	28.16	28.20	28.09	28.17	28.21	28.23	**28.29**
	**SSIM**	0.7583	0.7552	0.7655	0.7587	0.7656	0.7605	0.7637	0.7658	0.7667	**0.7683**
	**SCC**	0.2625	0.2529	0.2761	0.2700	0.2738	0.2632	0.2751	0.2760	0.2769	**0.2793**
	**SAM**	0.1038	0.1046	0.1018	0.1038	0.1028	0.1031	0.1013	0.1018	0.1015	**0.1004**
	**UQI**	0.9913	0.9912	0.9917	0.9914	0.9917	0.9914	0.9917	0.9917	0.9917	**0.9921**
**×3**	**P** **S** **NR**	26.13	26.31	26.30	26.23	26.35	26.21	26.30	26.31	26.38	**26.52**
	**SSIM**	0.6528	0.6634	0.6625	0.6622	0.6650	0.6558	0.6615	0.6655	0.6669	**0.6714**
	**SCC**	0.1341	0.1487	0.1491	0.1484	0.1511	0.1368	0.1463	0.1535	0.1539	**0.1552**
	**SAM**	0.1301	0.1276	0.1278	0.1302	0.1271	0.1290	0.1278	0.1276	0.1266	**0.1230**
	**UQI**	0.9865	0.9870	0.9871	0.9864	0.9871	0.9866	0.9871	0.9869	0.9872	**0.9883**
**×4**	**P** **S** **NR**	25.12	25.16	25.26	25.13	25.32	25.24	25.26	25.30	25.35	**25.48**
	**SSIM**	0.5912	0.5902	0.5988	0.5898	0.6028	0.5966	0.6029	0.6013	0.6040	**0.6093**
	**SCC**	0.0735	0.0717	0.0841	0.0669	0.0862	0.0782	0.0881	0.0846	0.0885	**0.0891**
	**SAM**	0.1461	0.1455	0.1440	0.1461	0.1429	0.1441	0.1438	0.1433	0.1425	**0.1403**
	**UQI**	0.9829	0.9831	0.9835	0.9829	0.9837	0.9834	0.9832	0.9836	0.9838	**0.9856**

**Table 2:** The performance of the proposed model on the AID dataset. The best results are highlighted in bold. The units for PSNR and SAM are dB and rad, respectively.

Scale	Metric	VDSR	SRDD	HSENet	TransENet	FENet	LGCNet	OmniSR	BSRAW	ASID	MSFANet
**×2**	**P** **S** **NR**	34.16	35.17	34.88	35.24	35.19	35.03	35.21	35.47	35.56	**35.84**
	**SSIM**	0.9247	0.9354	0.9319	0.9344	0.9355	0.9340	0.9357	0.9364	0.9364	**0.9371**
	**SCC**	0.5941	0.6412	0.6250	0.6398	0.6404	0.6346	0.6418	0.0642	0.6432	**0.6451**
	**SAM**	0.0609	0.0548	0.0567	0.0573	0.0547	0.0557	0.0546	0.0542	0.0541	**0.0536**
	**UQI**	0.9959	0.9968	0.9966	0.9966	0.9968	0.9967	0.9968	0.9969	0.9969	**0.9971**
**×3**	**P** **S** **NR**	30.18	31.04	30.95	30.89	31.12	30.69	31.02	31.16	31.30	**31.37**
	**SSIM**	0.8311	0.8521	0.8492	0.8523	0.8542	0.8449	0.8517	0.8555	0.8581	**0.8594**
	**SCC**	0.3408	0.3939	0.3886	0.3789	0.3972	0.3738	0.3911	0.4013	0.4086	**0.4131**
	**SAM**	0.0942	0.0856	0.0865	0.0898	0.0848	0.0887	0.0859	0.0843	0.0830	**0.0813**
	**UQI**	0.9916	0.9929	0.9928	0.9924	0.9930	0.9923	0.9929	0.9930	0.9933	**0.9934**
**×4**	**P** **S** **NR**	28.13	28.89	28.74	28.41	28.94	28.89	28.89	28.94	29.05	**29.10**
	**SSIM**	0.7521	0.7779	0.7724	0.7603	0.7798	0.7788	0.7781	0.0787	0.7841	**0.7848**
	**SCC**	0.2086	0.2643	0.2517	0.2578	0.2665	0.2646	0.2628	0.2683	0.2739	**0.2745**
	**SAM**	0.1173	0.1080	0.1097	0.1103	0.1069	0.1077	0.1080	0.1072	0.1058	**0.1055**
	**UQI**	0.9873	0.9892	0.9890	0.9881	0.9894	0.9893	0.9893	0.9893	0.9896	**0.9898**

**Table 3:** The performance of the proposed model on the WHU-RS19 dataset. The best results are highlighted in bold. The units for PSNR and SAM are dB and rad, respectively.

Scale	Metric	VDSR	SRDD	HSENet	TransENet	FENet	LGCNet	OmniSR	BSRAW	ASID	MSFANet
**×2**	**P** **S** **NR**	30.36	30.87	30.03	30.91	30.95	30.58	30.92	30.67	30.97	**31.04**
	**SSIM**	0.8369	0.8488	0.8302	0.8514	0.8515	0.8421	0.8505	0.8440	0.8519	**0.8527**
	**SCC**	0.3279	0.3544	0.2934	0.3628	0.3610	0.3373	0.3578	0.3427	0.3614	**0.3657**
	**SAM**	0.0827	0.0786	0.0866	0.0781	0.0778	0.0806	0.0782	0.0804	0.0776	**0.0772**
	**UQI**	0.9891	0.9901	0.9887	0.9896	0.9893	0.9893	0.9902	0.9897	0.9904	**0.9905**
**×3**	**P** **S** **NR**	27.76	28.19	27.96	28.22	28.15	27.99	28.23	28.11	28.28	**28.34**
	**SSIM**	0.7400	0.7543	0.7445	0.7565	0.7530	0.7451	0.7541	0.7543	0.7565	**0.7590**
	**SCC**	0.1837	0.2077	0.1953	0.2150	0.2061	0.1937	0.2091	0.2093	0.2119	**0.2169**
	**SAM**	0.1127	0.1079	0.1106	0.1072	0.1080	0.1095	0.1075	0.1109	0.1065	**0.1062**
	**UQI**	0.9827	0.9840	0.9833	0.9834	0.9836	0.9833	0.9842	0.9832	0.9846	**0.9847**
**×4**	**P** **S** **NR**	25.93	26.59	26.51	26.63	26.47	26.46	26.62	26.69	26.70	**26.75**
	**SSIM**	0.6505	0.6812	0.6780	0.6860	0.6819	0.6770	0.6818	0.6855	0.6872	**0.6873**
	**SCC**	0.0943	0.1326	0.1296	0.1398	0.1392	0.1257	0.1341	0.1393	0.1398	**0.1416**
	**SAM**	0.1402	0.1302	0.1313	0.1291	0.1307	0.1308	0.1298	0.1285	0.1283	**0.1280**
	**UQI**	0.9751	0.9783	0.9779	0.9770	0.9779	0.9779	0.9784	0.9788	0.9791	**0.9793**

**Table 4:** Ablation study of the proposed model at 4×factor. The best results are highlighted in bold.

Scenarios	Baseline+	Parameter (M)	Memory (MB)	RSSCN7		AID		WHU-RS19	
				PSNR	SSIM	PSNR	SSIM	PSNR	SSIM
**L0**	**——**	1.37	5.26	24.84	0.5738	27.64	0.7308	25.95	0.6494
**L1**	**FRA**	1.36	5.23	24.83	0.5761	27.74	0.7348	25.91	0.6483
**L2**	**LSO**	0.64	3.6	24.86	0.5761	27.71	0.7324	25.99	0.6515
**L3**	**RFN**	8.93	12.10	25.07	0.5887	27.63	0.7318	26.28	0.6683
**L4**	**FRA+LSO**	0.94	3.64	24.85	0.5761	27.69	0.7322	25.98	0.6512
**L5**	**FRA+RFN**	8.92	12.05	25.03	0.5859	27.64	0.7317	26.21	0.6642
**L6**	**LSO+RFN**	10.61	14.22	25.31	0.6017	28.82	0.7766	26.69	0.6858
**L7**	**MSFANet**	**11.98**	**16.3**	**25.48**	**0.6093**	**29.1**	**0.7848**	**26.75**	**0.6873**

**Table 5:** Performance of MSFANet with different window sizes and attention heads on the AID dataset (×4 super-resolution task). The best results are highlighted in bold.

Model	Window Size	Attention Heads	AID		WHU-RS19	
			PSNR	SSIM	PSNR	SSIM
**MSFANet**	**2 × 2**	**2**	28.87	0.7753	26.58	0.6821
		**4**	28.95	0.7786	26.62	0.6835
	**4 × 4**	**2**	29.03	0.7822	26.65	0.6848
		**4**	29.05	0.7832	26.70	0.6861
	**8 × 8**	**2**	29.02	0.7805	26.68	0.6853
		**4**	**29.1** **0**	**0.78** **48**	**26.7** **5**	**0.68** **73**
	**16 × 16**	**2**	29.08	0.7791	26.66	0.6842
		**4**	29.07	0.7818	26.69	0.6859

## Data Availability

The data and code are accessible at the following link: https://github.com/AlvinsaideYu/MSFANet (accessed on 19 September 2025).

## Supplementary Materials

The following supporting information can be downloaded at: https://www.mdpi.com/article/10.3390/s25216729/s1, Figure S1. Visual comparison of the proposed model and the SOTA methods on three test images from the AID dataset; Figure S2. Visual comparison of the proposed model and the SOTA methods on three test images from the WHU-RS19 dataset; Table S1. The performances on categories within the RSSCN7 dataset for the ×4 upscaling; Table S2. The performances on categories within the AID dataset for the ×4 upscaling; Table S3. The performances on categories within the WHU-RS19 dataset for the ×4 upscaling.
