# Supplementary material for: MSFANet: A Multi-Scale Feature Fusion Transformer with Hybrid Attention for Remote Sensing Image Super-Resolution
Source: Sensors (Basel). 2025 Nov 3;25(21):6729. doi: 10.3390/s25216729 (PMC12610848; doi:10.3390/s25216729)
Supplement: Supplementary file 1 [file sensors-25-06729-s001.zip › sensors-3914648-supplementary.pdf]

SUPPLEMENTARY INFORMATION

MSFANet: A Multi-Scale Feature Fusion Transformer with Hybrid Attention for Remote Sensing Image Super-Resolution

Jie Yu [1], Chengcheng Lin [1], Luyao Peng [1], Cheng Zhong [1] and Hui Li [2,*]

[1] Badong National Observation and Research Station of Geohazards, China University of Geosciences, Wuhan 430074, China; jieyu@cug.edu.cn (J.Y.); linchengcheng@cug.edu.cn (C.L.); luyaopeng@cug.edu.cn (L.P.); zhonglxm@cug.edu.cn (C.Z.)
[2] The School of Earth Sciences, China University of Geosciences, Wuhan 430074, China
* Correspondence: rslihui@cug.edu.cn

This file includes:

Tables S1–S3

Figures S1 and S2

TABLE S1

The performances on categories within the RSSCN7 dataset for the ×4 upscaling. The best results are highlighted in red, the second best in blue, and the third best in green, respectively

Categories	TransENet		FENet		LGCNet		OmniSR		BSRAW		ASID		MSFANet	
	PSNR	SSIM	PSNR	SSIM	PSNR	SSIM	PSNR	SSIM	PSNR	SSIM	PSNR	SSIM	PSNR	SSIM
**Grasslands**	28.07	0.6590	28.28	0.6667	28.18	0.6621	28.24	0.6651	28.15	0.6661	28.30	0.6671	28.34	0.6756
**Forests**	30.43	0.6251	30.55	0.6278	30.49	0.6252	30.53	0.6277	30.49	0.6277	30.58	0.6285	30.63	0.6327
**Farmland**	23.37	0.6116	23.59	0.6301	23.50	0.6223	23.57	0.6281	23.62	0.6325	23.67	0.6331	23.87	0.6352
**Parking lots**	26.52	0.7211	26.77	0.7321	26.65	0.7252	26.74	0.7311	26.69	0.7305	26.80	0.733	26.94	0.7362
**Village**	24.5	0.4647	24.60	0.4727	24.57	0.4695	24.58	0.4726	24.49	0.4728	24.60	0.4752	24.77	0.4738
**Factory**	21.37	0.5294	21.61	0.5504	21.52	0.5414	21.59	0.5481	21.57	0.5501	21.65	0.5513	21.88	0.5787
**Rivers**	21.66	0.5188	21.85	0.5397	21.78	0.5308	21.83	0.537	21.84	0.5407	21.87	0.5403	21.94	0.566
**Average**	25.13	0.5899	25.32	0.6028	25.24	0.5966	25.30	0.601	25.26	0.6029	25.35	0.6040	25.48	0.61

TABLE S2

The performances on categories within the AID dataset for the ×4 upscaling. The best results are highlighted in red, the second best in blue, and the third best in green, respectively.

Categories	TransENet		FENet		LGCNet		OmniSR		BSRAW		ASID		MSFANet	
	PSNR	SSIM	PSNR	SSIM	PSNR	SSIM	PSNR	SSIM	PSNR	SSIM	PSNR	SSIM	PSNR	SSIM
**Airport**	28.75	0.782	29.31	0.7979	29.22	0.796	29.25	0.7968	29.29	0.798	29.41	0.801	29.44	0.8015
**Bareland**	38.11	0.9003	38.27	0.9027	38.21	0.9022	38.28	0.9028	38.25	0.9025	38.26	0.9031	38.3	0.9032
**Baseballfield**	29.95	0.8432	30.54	0.857	30.6	0.857	30.51	0.8556	30.62	0.8581	30.84	0.8612	30.85	0.8616
**Beach**	32.13	0.8388	32.46	0.8437	32.44	0.8441	32.45	0.8434	32.44	0.8437	32.49	0.8449	32.55	0.8451
**Bridge**	29.69	0.7848	30.27	0.7979	30.10	0.7955	30.18	0.7965	30.23	0.7977	30.35	0.7999	30.41	0.8008
**Center**	26.63	0.7107	27.45	0.7451	27.39	0.7417	27.44	0.7423	27.48	0.7459	27.62	0.7502	27.67	0.7532
**Church**	25.37	0.7457	26.16	0.7762	26.05	0.7741	26.09	0.7742	26.17	0.778	26.29	0.7816	26.31	0.7819
**Commercial**	27.88	0.7616	28.26	0.7779	28.19	0.7753	28.22	0.7759	28.28	0.7787	28.36	0.7818	28.4	0.7826
**Denseresidential**	23.3	0.6359	23.92	0.6741	23.86	0.6718	23.86	0.6709	23.95	0.6778	24.03	0.6824	24.07	0.6831
**Desert**	37.23	0.9121	37.37	0.9146	37.27	0.9142	37.40	0.9147	37.2	0.9145	37.35	0.9142	37.44	0.9153
**Farmland**	31.57	0.7781	32.05	0.7918	31.95	0.7898	32.01	0.7907	32.04	0.7917	32.15	0.7949	32.18	0.7956
**Forest**	28.63	0.7066	28.90	0.7217	28.91	0.7241	28.86	0.72	28.87	0.7225	28.94	0.7256	28.92	0.7241
**Industrial**	27.58	0.779	28.07	0.7975	28.06	0.7967	28.03	0.7963	28.10	0.799	28.21	0.8025	28.24	0.8041
**Meadow**	32.73	0.7281	32.83	0.7314	32.83	0.7314	32.81	0.7312	32.84	0.7313	32.87	0.7323	32.88	0.7324
**Mediumresidential**	28.16	0.7222	28.90	0.7526	28.81	0.7518	28.82	0.7504	28.91	0.7555	29.0	0.7596	29.07	0.7599
**Mountain**	30.51	0.7829	30.66	0.7901	30.69	0.7909	30.65	0.7893	30.66	0.7904	30.72	0.7924	30.84	0.793
**Park**	27.65	0.7687	27.90	0.7824	27.93	0.7833	27.90	0.7814	27.89	0.7826	28.01	0.7872	28.03	0.7878
**Parking**	25.41	0.8277	26.78	0.8658	26.63	0.8633	26.60	0.8636	26.83	0.8688	27.18	0.8755	27.3	0.8771
**Playground**	32.85	0.8969	34.09	0.9189	34.06	0.9175	33.97	0.9169	34.04	0.9184	34.25	0.9207	34.37	0.9221
**Pond**	30.05	0.8348	30.56	0.8454	30.55	0.8452	30.51	0.8443	30.54	0.8455	30.69	0.8483	30.7	0.8489
**Port**	26.38	0.8217	27.01	0.8446	26.94	0.8432	26.96	0.8425	27.01	0.8445	27.12	0.8481	27.16	0.8488
**Railwaystation**	27.07	0.7335	27.71	0.7597	27.63	0.7569	27.64	0.7566	27.67	0.7596	27.78	0.7624	27.83	0.7651
**Resort**	26.42	0.711	26.91	0.7311	26.89	0.7315	26.87	0.7299	26.94	0.7331	27.02	0.7364	27.06	0.7368
**River**	30.55	0.7526	30.79	0.7601	30.78	0.7596	30.79	0.7597	30.81	0.7606	30.86	0.7624	30.89	0.7628
**School**	25.13	0.7281	25.59	0.7517	25.57	0.7527	25.55	0.7498	25.63	0.7545	25.76	0.7604	25.87	0.7638
**Sparseresidential**	25.3	0.6012	25.51	0.6155	25.51	0.6174	25.50	0.6156	25.52	0.6182	25.57	0.6217	25.55	0.6198
**Square**	24.78	0.7039	25.48	0.7364	25.46	0.7356	25.41	0.7336	25.52	0.738	25.66	0.7444	25.68	0.7451
**Stadium**	24.12	0.7149	24.84	0.7471	24.79	0.7447	24.80	0.7447	24.86	0.7483	24.98	0.7532	25.02	0.7539
**Storagetanks**	23.84	0.6724	24.38	0.7036	24.32	0.7013	24.32	0.7001	24.40	0.7047	24.47	0.7087	24.49	0.7097
**Viaduct**	24.81	0.6302	25.35	0.6594	25.30	0.6565	25.26	0.6551	25.34	0.6592	25.48	0.6665	25.49	0.6675
**Average**	28.41	0.7603	28.94	0.7797	28.89	0.7788	28.89	0.7781	28.95	0.7807	29.05	0.7841	29.10	0.7848

TABLE S3

The performances on categories within the WHU-RS19 dataset for the ×4 upscaling. The best results are highlighted in red, the second best in blue, and the third best in green, respectively

Categories	TransENet		FENet		LGCNet		OmniSR		BSRAW		ASID		MSFANet	
	PSNR	SSIM	PSNR	SSIM	PSNR	SSIM	PSNR	SSIM	PSNR	SSIM	PSNR	SSIM	PSNR	SSIM
Desert	36.14	0.88	36.3	0.8798	35.74	0.8775	36.38	0.8805	36.35	0.8808	36.28	0.8798	36.41	0.8811
Farmland	31.27	0.7333	31.16	0.7261	31.16	0.7275	31.3	0.7307	31.35	0.7327	31.39	0.7341	31.4	0.7352
Mountain	21.87	0.4752	21.8	0.4679	21.86	0.4733	21.87	0.4725	21.88	0.4742	21.92	0.4783	21.9	0.4761
Park	25.33	0.6135	25.19	0.6036	25.28	0.6068	25.31	0.6092	25.37	0.6115	25.41	0.6146	25.41	0.6142
Airport	24.94	0.6861	24.56	0.6659	24.76	0.6749	24.86	0.6801	24.93	0.6844	24.98	0.6868	25.02	0.6884
Beach	36.09	0.9319	35.95	0.9289	35.4	0.9281	36.26	0.9316	36.27	0.9324	36.28	0.932	36.38	0.933
Bridge	30.41	0.8431	29.85	0.8325	30.27	0.8379	30.36	0.8413	30.58	0.8447	30.52	0.8449	30.61	0.8453
Commercial	22.21	0.5924	21.95	0.5729	22.11	0.5834	22.18	0.5875	22.22	0.591	22.24	0.5935	22.25	0.5937
FootballField	25.79	0.719	25.32	0.697	25.61	0.7061	25.74	0.7137	25.81	0.7172	25.89	0.7205	25.85	0.7201
Forest	24.85	0.4655	24.78	0.456	24.83	0.4616	24.87	0.4641	24.88	0.4658	24.91	0.4703	24.89	0.4668
Industrial	24.39	0.6762	23.94	0.6469	24.32	0.6628	24.41	0.6704	24.54	0.676	24.54	0.6773	24.57	0.6778
Meadow	31.73	0.698	31.77	0.6971	31.74	0.6963	31.80	0.6982	31.81	0.6981	31.82	0.6986	31.83	0.6989
Parking	23.37	0.7302	22.64	0.6928	23.07	0.716	23.16	0.7196	23.44	0.7296	23.34	0.7316	23.48	0.7317
Pond	28.65	0.8117	28.5	0.8046	28.56	0.8074	28.67	0.81	28.73	0.8123	28.75	0.8127	28.78	0.8133
Port	25.36	0.7756	24.9	0.7574	25.18	0.7599	25.28	0.773	25.33	0.776	25.38	0.7791	25.42	0.7779
RailwayStation	23.27	0.5635	22.79	0.5288	23.1	0.5465	23.2	0.554	23.32	0.5609	23.34	0.5626	23.41	0.5668
Residential	22.63	0.6525	22.23	0.6253	22.45	0.6396	22.57	0.6467	22.67	0.6522	22.65	0.6531	22.68	0.6536
River	25.47	0.5945	25.34	0.5867	25.41	0.5862	25.45	0.5919	25.49	0.5942	25.53	0.5965	25.5	0.595
Viaduct	22.25	0.5917	21.74	0.5549	22.04	0.5727	22.14	0.5799	22.29	0.5917	22.26	0.5912	22.32	0.594
Average	26.63	0.686	26.45	0.6697	26.46	0.677	26.62	0.6818	26.70	0.6855	26.70	0.6872	26.75	0.6873

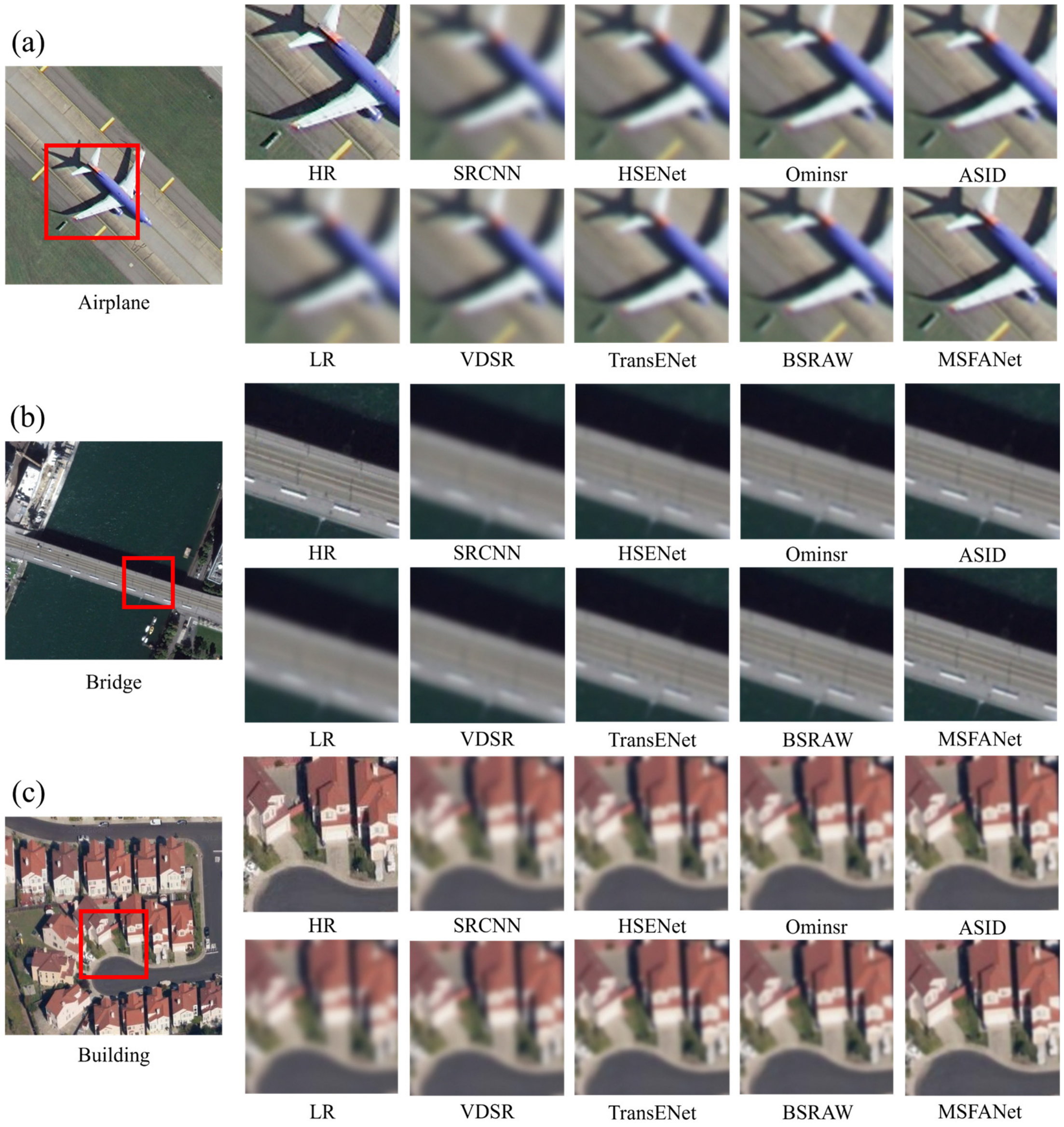

Figure S1. Visual comparison of the proposed model and the SOTA methods on three test images from the AID dataset. (a)Airplane, (b)Bridge, (c)Building.

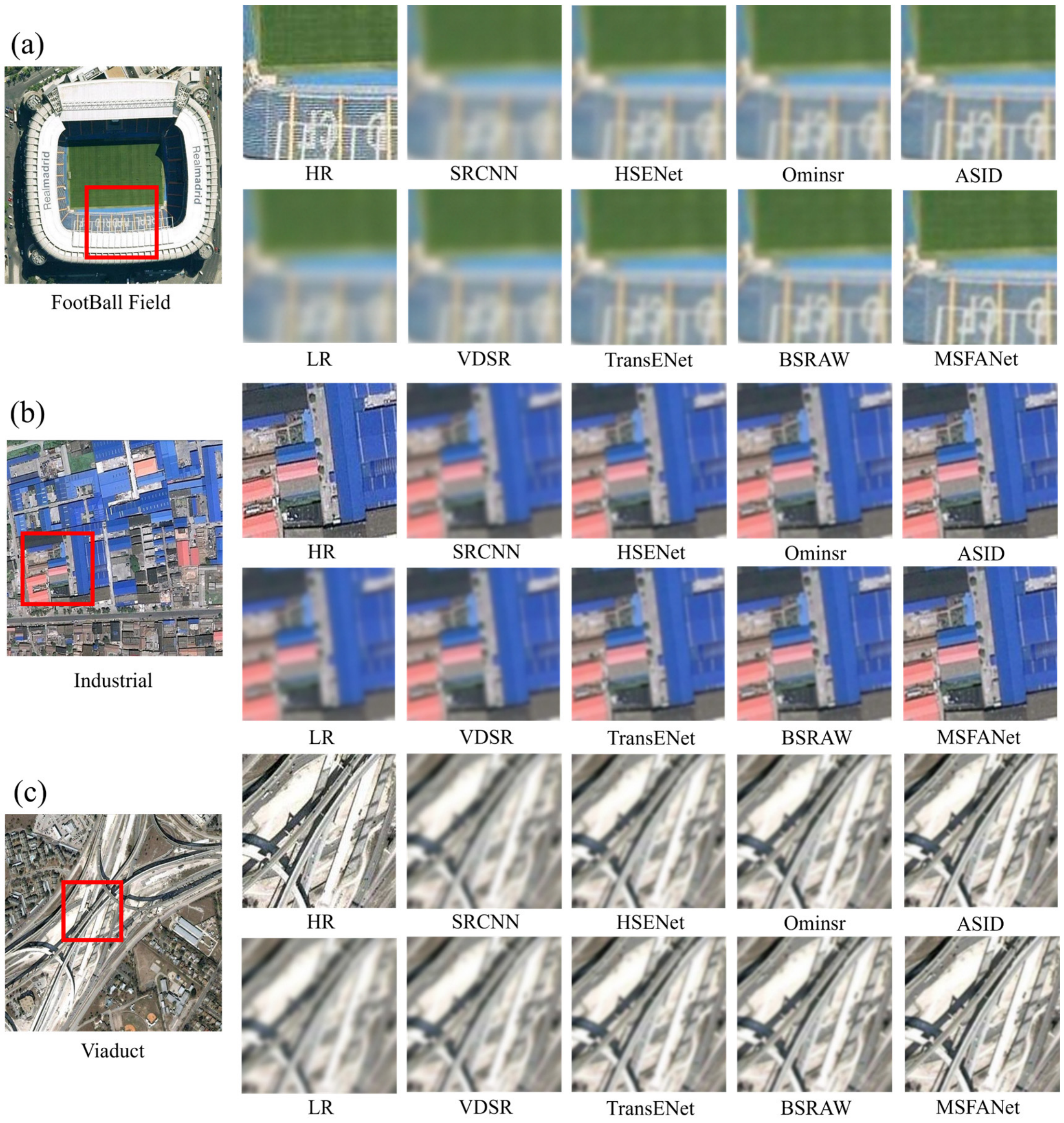

Figure S2.Visual comparison of the proposed model and the SOTA methods on three test images from the WHU-RS19 dataset. (a)FootBall Field, (b)Industrial, (c)Viaduct.
